# Supplementary material for: Identifying myocardial injuries in “normal-appearing” myocardium in pediatric patients with clinically suspected myocarditis using mapping techniques
Source: PeerJ. 2020 Nov 4;8:e10252. doi: 10.7717/peerj.10252 (PMC7648451; doi:10.7717/peerj.10252)
Supplement: Supplemental Information 2 [file peerj-08-10252-s002.docx]

1. Group 1:normal control; Group 2:subacute/chronic myocarditis; Group 3:acute myocarditis
2. Sex 1: male; Sex2: female
3. HCT: hematocrit
4. HR: Heart rate
5. BSA: Body Surface Area
6. EDV: end-diastolic volume;
7. ESV: end-systolic volume;
8. LVM: LV mass;
9. SV: stroke volume;
10. LVEF: left ventricle ejection fraction.
11. HLA: horizontal long axis;
12. SA: short axis;
13. ECV: extracellular volume;
